# High-Flow Nasal Cannula versus Noninvasive Positive Pressure Ventilation in Patients with Heart Failure after Extubation: An Observational Cohort Study

**DOI:** 10.1155/2020/6736475

**Published:** 2020-07-03

**Authors:** Che-Jung Chang, Ling-Ling Chiang, Kuan-Yuan Chen, Po-Hao Feng, Chien-Ling Su, Han-Shui Hsu

**Affiliations:** ^1^Institute of Emergency and Critical Care Medicine, National Yang-Ming University, Taipei, Taiwan; ^2^Division of Pulmonary Medicine, Department of Internal Medicine, Shuang Ho Hospital, Taipei Medical University, Taipei, Taiwan; ^3^School of Respiratory Therapy, College of Medicine, Taipei Medical University, Taipei, Taiwan; ^4^Division of Pulmonary Medicine, Department of Internal Medicine, School of Medicine, College of Medicine, Taipei Medical University, Taipei, Taiwan

## Abstract

Noninvasive positive pressure ventilation (NPPV) has been widely applied in patients with high-risk extubation failure, including heart failure. High-flow nasal cannula (HFNC) has been demonstrated to benefit patients with heart failure by reducing cardiac preload. This study aimed to compare the effectiveness of HFNC to NPPV for preventing extubation failure in patients with heart failure. This 3-year retrospective and single-center cohort study included patients with heart failure with left ventricular ejection fraction <50% who received prophylactic HFNC or NPPV after scheduled extubation from January 2015 to January 2018 from a medical center with four adult intensive care units. Demographics, comorbidities, diagnosis, and weaning status were collected. The primary outcome was treatment failure within 72 hours after extubation, which was defined as escalation to NPPV or reintubation in the HFNC group and was defined as requiring reintubation in the NPPV group. Secondary outcomes were reintubation within 72 hours, reintubation, duration of stay, and mortality during the intensive care unit and hospital stay. Of the 104 patients analyzed, characteristics of 58 patients in the HFNC group and 46 patients in the NPPV group were compared. The treatment failure within 72 hours in the two groups was not significantly different (25.9% vs 13%, *p*=0.106). Hypoxemic respiratory failure related treatment failure was significantly higher in the HFNC group. Prophylactic HFNC as first-line therapy had a comparable rate of reintubation within 72 hours to the prophylactic NPPV alone (17.2% vs 13%, *p*=0.556). Other secondary outcomes were similar between the two groups. Among patients with heart failure, HFNC was not inferior to NPPV for preventing extubation failure and reintubation. However, in case of an impending respiratory failure, selective patients may benefit from rescue NPPV.

## 1. Introduction

The incidence of extubation failure and reintubation in critically ill patients is approximately 10% to 30%, which leads to high mortality rate after weaning from mechanical ventilation (MV) [1, 2]. Studies showed that not only preexisting heart failure but also cardiac etiology for respiratory failures such as systolic heart failure, acute coronary syndrome, and severe valvular heart disease were high risks for extubation failure [3, 4]. Moreover, cardiac function and left ventricular ejection fracture (LVEF) were strongly associated with extubation failure [5]. Therefore, in the postextubation period in which cardiac performance is still in recovery, reducing the work of the cardiopulmonary system to prevent extubation failure in patients with heart failure is extremely important.

Positive end-expiratory pressure (PEEP) during invasive mechanical ventilation is one of the nonpharmacological approaches beneficial for heart failure caused by an increase in the intrathoracic pressure [6]. First, PEEP can augment cardiac output through a decrease in the right ventricular preload, left ventricular preload, and afterload [6]. Second, PEEP can counterbalance the hydrostatic force and decrease the extravascular lung water by exerting pressure at the level of the alveoli and interstitium [6]. Third, PEEP helps maintain alveolar pressure to prevent alveolar collapse [7]. However, end positive airway pressure (EPAP) is used for noninvasive ventilation and generates hemodynamic effects in cases of heart failure, similar to continuous positive airway pressure (CPAP) delivered by a face mask [7], which increases cardiac performance and stroke volume in patients with heart failure and a high left ventricular filling pressure [8–10]. Two noninvasive respiratory devices can generate positive pressure during the expiratory phase and have been used to prevent extubation failure, namely, high-flow nasal cannula (HFNC) and noninvasive positive pressure ventilation (NPPV).

HFNC is characterized by the stable concentration of oxygen and variable PEEP, which might have potential benefits for patients with heart failure [11]. A study found that HFNC could reduce the inspiratory inferior vena cava (IVC) collapse and right heart preload [12]. Further, HFNC could relieve dyspnea and hypoxemia in cardiogenic pulmonary edema [13, 14].

In the past decade, NPPV was extensively used for preventing extubation failure in high-risk patients, and this was proved with a moderate level of evidence [15, 16]. Two large randomized controlled trials demonstrated that NPPV could be applied for the prevention of postextubation respiratory failure in patients at risk of extubation failure, including those with heart failure [17, 18]. Recently, HFNC was increasingly used after scheduled extubation as an alternative therapy of NPPV because of its ease of application, patient tolerance, physiological benefits, and lower adverse events [19, 20]. Randomized studies showed that HFNC was not inferior to NPPV for preventing extubation failure in general high-risk extubation failure and postcardiothoracic surgery patients [21, 22].

However, in patients with heart failure, to the best of our knowledge, no studies have compared the potential benefits obtained with NPPV and those with HFNC after extubation. Therefore, this retrospective study aimed to compare the effectiveness of HFNC and NPPV for preventing extubation failure in critically ill patients with heart failure.

## 2. Materials and Methods

### 2.1. Study Design

A retrospective observational cohort study was conducted between January 2015 and January 2018 at a medical center with four adult intensive care units (ICUs) in New Taipei City, Taiwan. This study was approved by the Ethics Committee of Taipei Medical University (N201808011), and informed consent was waived because of its observational nature.

### 2.2. Patient Selection

**Figure 1:**
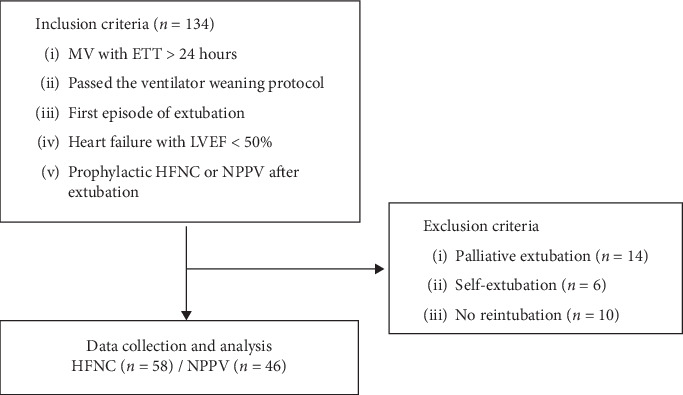
Retrospective flow chart of patients included in analyses between January 2015 and January 2018. ETT, endotracheal tube; HFNC, high-flow nasal cannula; MV, mechanical ventilation; NPPV, noninvasive positive pressure ventilation.

We reviewed a total of 5,497 ICU patients who had received MV for tracheal intubation and included 134 patients according to the following criteria: patients (1) who were on mechanical ventilator with endotracheal tube for more than 24 h, (2) who passed the weaning readiness assessment, (3) who had first episode of extubation, (4) who had heart failure with LVEF <50% determined by echocardiography under M-mode or Simpson's rule in the apical four-chamber view before extubation, and (5) on whom prophylactic HFNC or NPPV was applied as first-line therapy after extubation. The exclusion criteria were tracheostomy, ventilator dependence status before admission, self-extubation, palliative extubation, and do-not-reintubate status. After implementing these criteria, a total of 104 patients were recruited for the analysis (Figure 1).

All patients have received the following standard ventilator bundle: (1) head and bed elevation at least 30° to 45°, (2) oral care with 0.12%–0.2% chlorhexidine at least twice a day, and (3) daily sedation interruption and screening for weaning from ventilator. The weaning readiness assessment evaluated by respiratory therapists and physicians included (1) recovery from the reason of intubation, (2) hemodynamic stability or administration of low-dose inotropic agent, (3) no sedation or low-dose sedation with RASS ≥ −1, (4) arterial blood gas sample, (5) weaning parameters, (6) spontaneous breathing trial (SBT), and (7) evaluation of airway patency and secretions.

Device selection after extubation followed a patient-oriented care concept. On the premise that noninvasive respiratory support devices (HFNC or NPPV) were available, we consistently applied these devices as postextubation preventive strategies for patients with heart failure and with LVEF <50%. Before extubation, respiratory therapists and intensivists would assess and decide which devices (HFNC or NPPV) should be applied to patients. The decision to provide HFNC depended on the following assessments: (1) risk of aspiration, (2) facial trauma, (3) claustrophobia, (4) excessive airway secretions or saliva that required suction twice per hour, and (5) patients could not tolerate mask ventilation and refuse it. If providing HFNC did not meet the above criteria, NPPV with stable positive airway pressure was first applied to patients as respiratory support after extubation.

### 2.3. Outcomes

Patients who received prophylactic HFNC as first-line therapy were compared with those who received prophylactic NPPV alone. The primary outcome was treatment failure within 72 hours after extubation. HFNC failure was defined as escalation to use of other respiratory devices including NPPV or invasive MV within 72 hours. NPPV failure was defined as the need for invasive MV within 72 hours. Secondary outcomes included reintubation within 72 hours, reintubation, ICU and hospital lengths of stay (LOS), and ICU and hospital mortality.

### 2.4. Data Collection

The following characteristics were collected: age, sex, body mass index (BMI), smoking status, Acute Physiology and Chronic Health Evaluation II (APACHE II) score upon ICU admission, echocardiographic data, diagnosis at admission, comorbidities, weaning status (includes rapid shallow breathing index, maximum inspiratory pressure, maximum expiratory pressure, cuff leak test, MV days before extubation, Glasgow coma scale on extubation day), and the setting of HFNC and NPPV. Treatment failure was determined on the basis of arterial blood gas (ABG) data and/or symptoms reported in the medical records and classified as follows: (1) hypoxemic respiratory failure, defined as peripheral capillary oxygen saturation (SpO_2_) < 90% or arterial partial pressure of oxygen (PaO_2_) < 60 mmHg with fraction of inspired oxygen (FiO_2_) > 0.5; (2) hypercapnic respiratory failure, defined as pH < 7.30 with arterial partial pressure of carbon dioxide (PaCO_2_) > 50 mmHg; (3) excessive effort for breathing, defined as respiratory rate > 35 breaths per minute and/or the use of accessory muscles and paradoxical respiration; (4) hemodynamic compromise, defined as cardiac arrest, bradycardia with loss of alertness, and/or severe hemodynamic instability; and (5) airway protection, defined as the persistent inability to remove respiratory secretions, upper airway obstruction, massive aspiration, and/or upper gastrointestinal bleeding. Pre-extubation ABG data for the patients with treatment failure were collected in order to confirm that they received prophylactic noninvasive respiratory devices under stable conditions. We determined the types of postextubation respiratory failure from the ABG data, with the exception of three types that could be identified by the findings of physical assessments mentioned in the medical records.

### 2.5. Statistical Analysis

Descriptive statistics were expressed as mean ± standard deviation (SD), median (Q1-Q3), or number (percentages). The chi-squared or Fisher's exact test was used to compare categorical variables. The Mann–Whitney *U* test was used to compare continuous variables between two independent samples. Additional univariate analyses for primary outcomes and multivariate logistic regression were performed to account for clinical confounding factors and statistical differences. We selected variables in the univariate analysis with *p* value < 0.20 and included them in the multivariate logistic regression. Considering the baseline differences between the groups, a propensity score was computed by using logistic regression with the baseline variables (baseline characteristics, echocardiography data, comorbidities, weaning parameters, and diagnosis). A matching method was performed using the propensity scores. A two-tailed *p* value < 0.05 was considered significant. Statistical analyses were performed using the statistical software IBM SPSS version 22 (IBM Corp., Armonk, NY, USA) and R statistical package (R Foundation for Statistical Computing, Vienna, Austria).

## 3. Results

A total of 104 patients were enrolled and analyzed in this study, including 58 in the HFNC group and 46 in the NPPV group. Clinical characteristics, echocardiographic data, diagnosis, comorbidities, disease severity, and weaning status in the two groups were similar. Among all patients, the median LVEF was 34.1% (27.3%–41.2%) and 72.1% of the patients were classified as having systolic heart failure (LVEF < 40%). The most common diagnosis upon admission was pneumonia, acute decompensated heart failure with pulmonary edema, and extrapulmonary sepsis, which were also similar between the two groups (Table 1).

The primary outcome, i.e., treatment failure within 72 hours, occurred in 15 (25.9%) patients with HFNC and six (13%) patients with NPPV, and no significant difference was noted between the two groups (*p*=0.106). However, the two groups differed with regard to the type of treatment failure, with the rate of hypoxemic respiratory failure within 72 hours being significantly lower in the NPPV group than in the HFNC group (*p*=0.046) (Table 2). According to the ABG data, the cause of the five types of treatment failure could be deterioration of the primary disease or underlying disease or inadequate respiratory support (Supplement 1). Secondary outcomes, including reintubation within 72 hours, reintubation, ICU and hospital LOS, and ICU and hospital mortality, were not significantly different between the two groups (Table 2). Even after matching, there was no significant between-group difference in terms of treatment failure within 72 hours (*p*=0.664) (Supplement 2).

**Figure 2:**
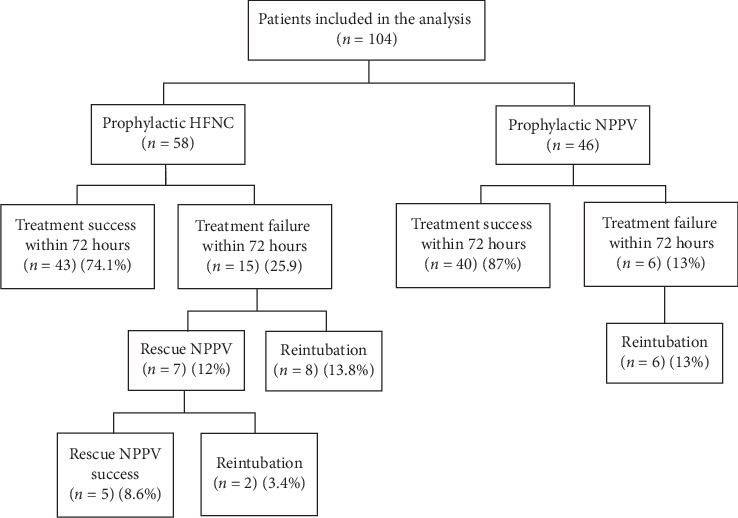
Outcomes following extubation within 72 hours. In the HFNC group, eight patients required immediate reintubation once treatment failure developed, and the other seven patients used NPPV as rescue therapy. Rescue NPPV successfully avoided reintubation in five patients, but it failed in two patients. In the NPPV group, six patients needed reintubation because of treatment failure. HFNC, high-flow nasal cannula; NPPV, noninvasive positive pressure ventilation.

**Figure 3:**
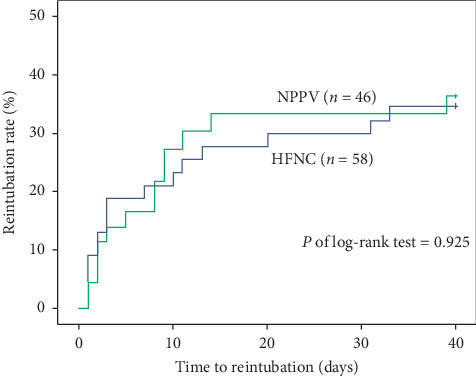
Kaplan–Meier plot of time to reintubation. HFNC, high-flow nasal cannula; NPPV, noninvasive positive pressure ventilation.

In the HFNC group, eight patients required immediate reintubation once treatment failure developed, and the other seven patients used NPPV as rescue therapy. Rescue NPPV successfully avoided reintubation in five patients, but it failed in two patients. In the NPPV group, six patients needed reintubation because of treatment failure (Figure 2). The reintubation rate of the NPPV group increased sharply 1 week after extubation, even if the reintubation rate was higher in HFNC previously. However, the reintubation rate was not significantly different between the two groups in the Kaplan–Meier plot (p of the log-rank test = 0.925) (Figure 3).

The results of the univariate analysis and multivariate logistic regression for variables associated with treatment failure within 72 hours are shown in Table 3. Variables in the univariate analysis with *p* value < 0.20 were HFNC vs NPPV (HFNC as first-line therapy compared with NPPV alone), admission diagnosis of pneumonia, and chronic kidney disease as underlying comorbidity. The above three variables were included in the multivariate logistic regression, and no independent variables were associated with treatment failure within 72 hours (Table 3).

## 4. Discussion

In this study, no significant difference exists between prophylactic HFNC as first-line therapy and prophylactic NPPV alone in preventing extubation failure in critically ill patients with heart failure.

We found that HFNC has been applied more in patients with heart failure than NPPV in the past 3 years because of its better comfort, patient tolerance, and easier assembly, which was different from the past practice of using prophylactic NPPV alone for such high-risk extubation failure population. Moreover, in this study, no significant difference was found between HFNC and NPPV in preventing treatment failure, which might be due to the following features of HFNC. First, the high-flow rate from the cannula against the expiratory airflow from patients generates the PEEP effect. Okuda et al. [23] confirmed the PEEP effect of HFNC by evaluating the correlation of end-expiratory esophageal pressure and flow rate. In this study, the initial flow of HFNC was 49.4 ± 6.15 L/min, and a previous study has shown that such high flow could generate a certain degree of PEEP [24]. Mauri et al. [25] demonstrated that HFNC setting greater than 40 L/min could improve dynamic lung compliance and reduce inspiratory effort and metabolic work of breathing, which represented external ventilation support. Roca et al. [12] found that the flow of HFNC increased and the IVC collapse decreased, which confirmed that HFNC reduced the cardiac preload. Furthermore, Makdee et al. [13] found that HFNC reduced the respiratory rate in patients with acute cardiogenic pulmonary edema.

Second, the heated and humidified flow facilitated secretion clearance [11]. In this study, 38% of the patients were admitted to the hospital due to pneumonia; hence, it was necessary to maintain airway clearance after extubation. Excessive secretion was one of the major causes of extubation failure [26], and HFNC might reduce sputum retention to improve outcomes for pneumonia patients [27]. A recent meta-analysis suggested that pneumonia patients might benefit from the physiological effects of HFNC, which could reduce ICU mortality [28]. In the present study, even though the multivariate logistic regression analysis showed that pneumonia tended to associate with treatment failure, HFNC was not inferior to NPPV as a prophylactic strategy as there are more pneumonia patients in the HFNC group.

Third, HFNC is characterized by higher patient comfort and tolerance; thus, its continued application was easier [22, 29]. In this study, HFNC was continuously administered to patients, but NPPV was administered intermittently due to patient compliance. Thus, NPPV was unable to provide sustained noninvasive respiratory support after extubation. Based on the above perspectives, we believe that HFNC might reduce the metabolism and load of respiratory muscles and provide adequate non-invasive respiratory support after extubation.

NPPV has been proven beneficial for heart failure by preventing alveolar collapse and counterbalancing alveolar fluids [30, 31]. Therefore, adequate threshold of PEEP and positive inspiratory pressure (PIP) supplied by NPPV might benefit some patients in this study. Three findings can support the above hypothesis. First, even if there was no significant difference in treatment failure between the HFNC and NPPV, treatment failure occurred half as often in the NPPV group. Furthermore, the incidence of hypoxemia respiratory failure was significantly lower in the NPPV group than in the HFNC group, which is consistent with the observation of our previous study that NPPV reduces postextubation respiratory failure caused by heart failure [26]. Previous studies also reported that better oxygenation with NPPV was probably due to higher PIP and PEEP compared with HFNC [21, 32]. Second, in the HFNC group, seven patients who received rescue NPPV had a worse cardiac function (median LVEF = 25.9%) than overall patients, but five of the seven patients were successfully weaned from the NPPV (Figure 2). Third, the reintubation rate increased sharply after 1 week in the NPPV group, which might be related to the withdrawal of NPPV after 72 hours (Figure 3). Accordingly, HFNC was similar to NPPV in preventing extubation failure, but NPPV might benefit selective patients with heart failure. Further research is needed to identify more accurate cardiac function indicators as standard for NPPV application.

A recent prospective study [33] reported that prophylactic NPPV reduced the 72-hour reintubation rate from 24% to 13% in patients aged >65 years and with underlying cardiac and respiratory disease, which was similar to our study population and reintubation rate. In our study, the reintubation rate within 72 hours in both groups (HFNC 17.2% vs NPPV 13%) were similar to that in Stéphan et al.'s study [21] (HFNC 14% vs NPPV 13.7%) and lower than those in Hernández et al.'s study [22] (HFNC 22.8% vs NPPV 19.1%). Even though our study included patients with more severe (median APACHE II = 22), worse cardiac function (median LEVF = 34.4%) and longer MV duration prior to extubation (median MV days = 9), the reintubation rate within 72 hours was lower than those in Hernández et al.'s study, which was due to two reasons. First, in the actual clinical setting, rescue NPPV was allowed when HFNC failed or postextubation respiratory failure occurred, and some patients benefited from rescue NPPV to reduce the reintubation rate. Second, the positive airway pressure and cardiopulmonary support provided by HFNC and NPPV might be beneficial to patients with cardiac dysfunction. Two randomized controlled trials demonstrated that NPPV reduces the rate of reintubation, which lead to a reduced ICU mortality in high-risk extubation patients [17, 18]. In this study, ICU mortality was relatively low in both devices, indicating the effectiveness of both in the period of postextubation. The hospital mortality rate was different between groups after the propensity score matching, but given the small sample size, further RCT is warranted.

The European Respiratory Society and American Thoracic Society clinical practice guidelines recommended that NPPV be used to prevent extubation failure in high-risk populations, despite the low certainty of evidence [34]. We could not exclude that NPPV is still an effective strategy for preventing extubation failure in patients with heart failure. However, from the perspective of preventing extubation failure, we considered that HFNC could be another alternative treatment for patients with underlying cardiac disease because of its ease of application and physiological benefits over conventional oxygen therapy [25], and our data demonstrated the effectiveness and safety of HFNC after extubation.

To our knowledge, no studies have investigated the effectiveness of HFNC and NPPV in critically ill patients with heart failure after extubation. The results of our data analysis were also consistent with our clinical experience. Nevertheless, this study has some limitations because of its retrospective nature. First, selection bias might not be completely avoided. Patients who were excluded from the analysis due to “palliative extubation” and “do not reintubation” status had poor outcomes, though the baseline characteristics were similar between the two cohorts. Second, the decision of using preventive respiratory devices was patient compliance-oriented, but there were no definite criteria for the assessment. Third, the clinical decision was in favor of applying HFNC to patients with excessive airway secretions that required frequent suctioning, and this may have caused bias. Fourth, the lack of physiological parameters directly confirmed the benefits of the two devices. Fifth, echocardiography was performed within a period before extubation, so it was difficult to accurately describe patients' cardiac function immediately during extubation. Besides, this study was conducted in one medical center with a small size sample. We look forward to a future prospective study or randomized controlled trial to explore the physiologic benefits and clinical outcomes of these two devices for patients with heart failure.

## 5. Conclusions

In critically ill patients with heart failure, there was no significant difference in the effectiveness of prophylactic HFNC in preventing extubation failure compared with NPPV. We suggest that prophylactic HFNC can be applied as first-line therapy after extubation. However, in case of an impending respiratory failure, selective patients may benefit from rescue NPPV.

## Figures and Tables

**Table 1:** Baseline characteristics of patients in the HFNC and NPPV groups.

Characteristics	HFNC (*n* = 58)	NPPV (*n* = 46)	*p* value
Age, years	74.5 (63.8–82.3)	72.5 (61.8–79)	0.366
Male, *n*	37 (63.8)	28 (60.9)	0.76
Smoke, *n*	24 (41.4)	18 (39.1)	0.816
BMI, kg/m^2^	23.03 (19.7–25.1)	23.39 (20.9–27.3)	0.214
APACHE II at ICU admission, point	21 (16.8–27)	24 (18–30)	0.074
Echocardiography			
LVEF, %	33.55 (26.6–41.2)	35.4 (27.9–41.1)	0.456
Systolic heart failure (LVEF < 40%), *n*	42 (72.4)	33 (71.1)	0.939
Moderate to severe VHD, *n*	26 (44.8)	25 (54.3)	0.335
Diagnosis of admission			
Cardiac arrest, *n*	4 (6.9)	3 (6.5)	>0.99
Pneumonia, *n*	25 (43.1)	15 (32.6)	0.275
Exacerbated COPD, *n*	1 (1.7)	2 (4.3)	0.582
Acute decompensated heart failure, *n*	11 (19)	14 (30.4)	0.174
Acute coronary syndrome, *n*	4 (6.9)	4 (8.7)	0.730
Extrapulmonary sepsis, *n*	8 (13.8)	4 (8.7)	0.419
Cardiac-thoracic surgery, *n*	1 (1.7)	2 (4.3)	0.582
Others: emergency surgery, *n*	1 (1.7)	2 (4.3)	0.582
Others, *n*	3 (5.2)	0	0.253
Comorbidities			
Hypertension, *n*	39 (67.2)	37 (80.4)	0.132
Coronary artery disease, *n*	27 (46.6)	19 (41.3)	0.593
Cerebrovascular disease, *n*	13 (22.4)	9 (19.6)	0.724
Obstructive lung disease, *n*	9 (15.5)	11 (23.9)	0.281
Diabetes mellitus, *n*	36 (62.1)	22 (47.8)	0.146
Chronic kidney disease, *n*	24 (41.1)	12 (26.1)	0.104
Cancer, *n*	6 (10.3)	3 (6.5)	0.728
Weaning status			
RSBI, cycles/min/L	59 (39.1–77.8)	74.65 (51.2–99.3)	0.055
Pimax, cmH2O	40 (36–55.5)	48 (40–64)	0.1
Pemax, cmH2O	50 (36–80)	68 (40–118)	0.135
Minute ventilation, L/min	9 (7.2–10.7)	8.33 (6.1–10.6)	0.371
Cuff leak test, ml	284 (184.8–352.5)	246 (131.5–346.5)	0.286
Duration of MV before extubation, days	9.5 (5.8–14)	8.5 (5–12)	0.539
GCS in the day of extubation, score	14 (13–15)	15 (13–15)	0.057
Initial setting of HFNC/NPPV			
HFNC flow, L/min	49.4 ± 6.15		
NPPV inspiratory pressure, cmH2O		13.43 ± 2.55	
NPPV PEEP level, cmH2O		7.3 ± 0.96	
Initial FiO_2_, %	41.91 ± 9.71	35.07 ± 4.94	0.449
The length of HFNC/NPPV use, hours	25.5 (20–68.5)	20.5 (2.75–71.75)	0.149

Values are mean ± SD, median (Q1-Q3), or number (percentage %). APACHE II, Acute Physiology and Chronic Health Evaluation II; BMI, body mass index; COPD, chronic obstructive pulmonary disease; FiO_2_, fraction of inspired oxygen; GCS, Glasgow Coma Scale; LVEF, left ventricular ejection fraction; HFNC, high-flow nasal cannula; MV, mechanical ventilation; NPPV, non-invasive positive pressure ventilation; PEEP, positive end expiatory pressure; Pemax, maximum expiratory pressure; Pimax, maximum inspiratory pressure; RSBI, rapid shallow breathing index; VHD, valvular heart disease ^*∗*^*p* value < 0.05.

**Table 2:** Outcomes of patients between the HFNC and NPPV groups.

Outcomes	HFNC (*n* = 58)	NPPV (*n* = 46)	*p* value
Primary outcome			
Treatment failure within 72 hours, *n*	15 (25.9)	6 (13)	0.106
Type of treatment failure			
Hypoxemia respiratory failure, *n*	11 (73.3)	1 (16.7)	0.046^∗^
Hypercapnia respiratory failure, *n*	3 (20)	2 (33.3)	0.598
Excessive effort for breathing, *n*	0	1 (16.7)	0.286
Cardiac arrest, *n*	1 (6.7)	0	>0.99
Airway protection, *n*	0	2 (33.3)	0.71
Time to treatment failure, hours	17 (4–54)	36 (4.5–49.8)	0.726

Secondary outcome			
Reintubation within 72 hours, *n*	10 (17.2)	6 (13)	0.556
Hospital reintubation, *n*	17 (29.3)	14 (30.4)	0.901
Time to reintubation, hours	69 (14–274)	145 (40.5–221.3)	0.691
ICU length of stay, days	14 (8–21.8)	11 (8–16.3)	0.138
Hospital length of stay, days	23.5 (16–46.3)	27 (18.5–38)	0.883
ICU mortality, *n*	1 (1.7)	1 (2.2)	>0.99
Hospital mortality, *n*	3 (5.2)	8 (17.4)	0.057

Values are mean ± SD, median (Q1–Q3), or number (percentage %); ICU, intensive care unit; HFNC, high-flow nasal cannula; NPPV, noninvasive positive pressure ventilation. ^*∗*^*p* value < 0.05.

**Table 3:** Univariate and multivariate analyses of variables associated with treatment failure and hospital mortality.

Variables	Treatment failure within 72 hours			
	Univariate analysis		Multivariate analysis	
	OR (95% CI)	*p* value	Adjusted OR (95% CI)	*p* value
Characteristics				
HFNC vs NPPV	2.326 (6.58–0.822)	0.112^§^	2.004 (0.687–5.841)	0.204
Reintubation	—		—	—
Age (years)	0.982 (0.952–1.014)	0.268	—	—
Male	0.969 (0.361–2.598)	0.95	—	—
Smoke	0.887 (0.332–2.371)	0.811	—	—
BMI, kg/m^2^	0.945 (0.863–1.034)	0.215	—	—
APACHE II at ICU admission, score	0.969 (0.906–1.037)	0.363	—	—

Echocardiography				
LVEF, %	1.011 (0.956–1.069)	0.704	—	—
Systolic heart failure	0.958 (0.331–2.771)	0.937	—	—
Moderate to severe VHD	1.182 (0.453–3.084)	0.732	—	—

Diagnosis of admission				
Cardiac arrest	—		—	—
Pneumonia	2.619 (0.986–6.956)^§^	0.053^§^	2.645 (0.968–7.227)	0.053
Exacerbated COPD	2.025 (0.175–23.463)	0.572	—	—
Acute decompensated heart failure	1.806 (0.634–5.143)	0.269	—	—
Acute coronary syndrome	—		—	—
Extrapulmonary sepsis	0.327 (0.04–2.689)	0.299	—	—

Comorbidities				
Hypertension	0.677 (0.241–1.904)	0.46	—	—
Coronary artery disease	0.564 (0.207–1.540)	0.264	—	—
Cerebrovascular disease	0.85 (0.254–2.843)	0.792	—	—
Obstructive lung disease	1.417 (0.449–4.471)	0.553	—	—
Diabetes mellitus	0.843 (0.323–2.201)	0.727	—	—
Chronic kidney disease	1.993 (0.753–5.278)^§^	0.165^§^	1.983 (0.718–5.475)	0.186

Weaning status				
RSBI, cycles/min/L	1.005 (0.99–1.021)	0.512	—	—
Pimax, cmH2O	1.008 (0.979–1.038)	0.6	—	—
Pemax, cmH2O	1.006 (0.992–1.021)	0.385	—	—
Minute ventilation, L/min	0.976 (0.836–1.139)	0.757	—	—
Cuff leak test, ml	1.0 (0.996–1.003)	0.958	—	—
MV days before extubation, days	1.036 (0.97–1.106)	0.293	—	—
GCS in the day of extubation, score	0.913 (0.731–1.139)	0.419	—	—

APACHE II, Acute Physiology and Chronic Health Evaluation II; BMI, body mass index; CI, confidence interval; COPD, chronic obstructive pulmonary disease; GCS, Glasgow Coma Scale; LVEF, left ventricular ejection fraction; HFNC, high-flow nasal cannula; MV, mechanical ventilation; NPPV, noninvasive positive pressure ventilation; OR, odds ratio; Pemax, maximum expiratory pressure; Pimax, maximum inspiratory pressure; RSBI, rapid shallow breathing index; VHD, valvular heart disease. ^§^*p* value < 0.2 in the univariate analysis. ^∗^*p* value < 0.05 in the multivariate analysis.

## Supplementary Materials

Supplementary Materials Supplement 1: arterial blood gas (ABG) data before extubation and at the time of treatment failure in patients with treatment failure. Supplement 2: baseline characteristics and outcomes between propensity score-matched patients in HFNC and NPPV groups.
