# Supplementary material for: High-Flow Nasal Cannula versus Noninvasive Positive Pressure Ventilation in Patients with Heart Failure after Extubation: An Observational Cohort Study
Source: Can Respir J. 2020 Jul 3;2020:6736475. doi: 10.1155/2020/6736475 (PMC7354657; doi:10.1155/2020/6736475)
Supplement: Supplementary Materials — Supplement 1: arterial blood gas (ABG) data before extubation and at the time of treatment failure in patients with treatment failure. Supplement 2: baseline characteristics and outcomes between propensity score-matched patients in HFNC and NPPV groups. [file 6736475.f1.pdf]

Supplementary Materials

Supplement 1. Arterial blood gas (ABG) data before extubation and at the time of treatment failure in patients with treatment failure

NO.	Group	Diagnosis	Type of treatment failure	Pre-extubation ABG								ABG at treatment failure							
				pH	$PaCO_2$	PaO_2	HCO_3^-	BE	SaO_2	FiO_2	PF ratio	PH	$PaCO_2$	PaO_2	HCO_3^-	BE	SaO_2	FiO_2	PF ratio
1	HFNC	Pneumonia	Hypoxemia	7.472	33.4	144.2	23.9	0.7	98.7	40	360.5	7.545	27.8	57.5	23.5	1.4	90.8	50	115
2 [a]	HFNC	ADHF	Hypoxemia						SpO_2 99	40		7.516	29.8	51.7	23.6	1.6	86.4	70	73.86
3	HFNC	Pneumonia	Hypoxemia	7.402	23.7	130.1	14.4	9.1	98.9	40	325.3	7.493	29	41.2	21.8	-0.9	73.6	60	68.67
4	HFNC	Pneumonia	Hypoxemia	7.5	33.2	73.5	25.3	2.5	96	30	245	7.49	34.4	53.3	26.1	3.1	87.8	60	88.83
5	HFNC	ADHF	Hypercapnia	7.379	51.9	132.6	29.9	4	59.5	40	331.5	7.32	57.1	63.9	28.8	1.9	59.5	70	91.29
6	HFNC	Pneumonia	Hypoxemia	7.387	27.2	166.3	16	-7.7	99	40	415.8	7.44	32.6	56.4	21.8	-1.7	88.2	50	112.8
7	HFNC	Pneumonia	Hypoxemia	7.502	38.2	140.6	29.3	5.7	98.7	40	351.5	7.445	40.3	67.1	24.8	-0.1	90.9	60	111.8
8 [a]	HFNC	Extrapulmonary Sepsis	Hypercapnia						SpO_2 99%	40		7.196	75.2	63.3	28.5	-1.4	89.7	70	90.43
9	HFNC	Pneumonia	Hypoxemia	7.457	41.1	103.6	28.4	4.2	98	30	345.3	7.46	40.3	38.7	28	3.9	75.8	70	55.29
10	HFNC	Pneumonia	Hypercapnia	7.422	38.6	174.8	24.6	0.2	99.5	40	437	7.326	77.3	53.1	39.5	10.6	83.4	85	62.47
11 [b]	HFNC	ADHF	Hypoxemia	7.361	38.1	303.5	21.1	-4	99.9	50	607						SpO_2 87%	60	
12	HFNC	Pneumonia	Hypoxemia	7.543	34	143	28.6	5.9	99.3	40	357.5	7.363	51.7	66.1	28.7	2.5	90.2	70	94.43
13	HFNC	Pneumonia	Hypoxemia	7.554	26.2	170	22.6	1.2	98.3	40	425	7.358	36.7	46.8	20.2	-4.7	81.1	60	78
14 [a]	HFNC	ADHF	Hypoxemia						SpO_2 98%	30		7.44	38.9	37.7	26.2	2.1	70.8	60	62.83
15	HFNC	ADHF	Cardiac arrest	7.438	36	204	23.8	-0.1	98.3	40	510	7.477	36.7	133	26.5	2.9	99.1	85	156.5

16 [c]	NPPV	ADHF	Hypoxemia	7.45	57.5	91.9	39.1	13	96.6	40	229.8						SpO_2 89%	60	
17	NPPV	Pneumonia	Excessive effort for breathing	7.343	42.6	138.4	22.6	-3	98.4	40	346	7.325	42.7	130.7	21.8	-4.1	98.4	40	326.8
18	NPPV	Pneumonia	Hypercapnia	7.445	43.6	95.1	29.3	4.8	98.1	30	317	7.306	74.2	61.4	36.2	8.3	86.7	30	204.7
19	NPPV	COPD	Hypercapnia	7.409	47.1	95.2	29.1	3.8	98.1	40	238	7.333	71.6	79.1	37.2	8.8	96.6	40	197.8
20	NPPV	ADHF	Airway protection	7.479	51.6	106.5	37.5	2	98.3	40	266.3	7.384	67.9	63.4	39.6	11.7	92.3	40	158.5
21	NPPV	Pneumonia	Airway protection	7.496	30.2	140.9	22.8	0	99	35	402.6	7.476	24.2	120.1	17.5	-5	98.4	40	300.3

HFNC, high-flow nasal cannula; NPPV, non-invasive positive pressure ventilation; ADHF, acute decompensated heart failure; COPD, chronic obstructive pulmonary disease; $PaCO_2$, arterial partial pressure of carbon dioxide; PaO_2, arterial partial pressure of oxygen; $HCO3^-$, bicarbonate; BE, base excess; SaO_2, arterial oxygen saturation; SpO_2, *peripheral capillary oxygen saturation*; FiO_2, fraction of inspired oxygen; PF ratio, PaO_2/FiO_2 ratio

[a] There was no ABG data before extubation, although the oxygenation status of the patient was stable.

[b] The SpO_2 of the patient dropped to 87% within a few minutes after extubation. The patient was diagnosed with hypoxemic respiratory failure by the clinician and immediately intubated, so ABG data were not available.

[c] The SpO_2 of the patient dropped to 89% within a few minutes after extubation. The patient was diagnosed with hypoxemic respiratory failure by the clinician and immediately intubated, so ABG data were not available.

Supplement 2. Baseline characteristics and outcomes between propensity score-matched patients in HFNC and NPPV groups.

Variables	HFNC (n=22)	NPPV (n=22)	p value
Characteristics			
Age, years	75 (64-83)	76.5 (65-81)	0.888
Male, n	17 (77.3)	14 (63.6)	0.322
Smoke, n	12 (54.5)	8 (36.4)	0.226
BMI, kg/m^2	23.58 (21.26-27.26)	23.17 (18.47-26.01)	0.526
APACHE II at ICU admission, point	21.5 (17-28)	24 (20-28)	0.663
Duration of MV before extubation, days	10 (6-15)	9.5 (6-12)	0.878
GCS in the day of extubation, score	14 (11-15)	13.5 (11-15)	0.971
Echocardiography			
LVEF, %	35.4 (25.9-45.6)	36.8 (30-41.5)	0.963
HFrEF, n	13 (59.1)	14 (63.6)	0.757
Moderate to severe VHD, n	11 (50)	12 (54.5)	0.763
Pulmonary hypertension, n	3 (16.4)	4 (18.2)	>0.99
Comorbidities			
Hypertension, n	18 (81.8)	17 (77.3)	>0.99
Coronary artery disease, n	14 (63.6)	8 (36.4)	0.07
Cerebrovascular disease, n	5 (22.7)	5 (22.7)	>0.99
Obstructive lung disease, n	6 (27.3)	6 (27.3)	>0.99
Diabetes mellitus, n	15 (68.2)	13 (59.1)	0.531
Chronic kidney disease, n	10 (45.5)	9 (40.9)	0.761
Cancer, n	2 (9.1)	2 (9.1)	>0.99

Weaning parameters			
RSBI, cycles/min/L	70.65 (50-89)	71 (47-91)	0.787
Pimax, cmH2O	40 (28-48)	40 (32-48)	0.972
Pemax, cmH2O	42 (32-80)	46 (24-72)	0.878
Minute ventilation, L/min	9.22 (6.74-10.4)	8.47 (6.72-10.58)	0.833
Diagnosis of admission			
Cardiac arrest, n	1 (4.5)	0	>0.99
Pneumonia, n	8 (36.4)	10 (45.5)	0.54
Exacerbated COPD, n	1 (4.5)	0	>0.99
Acute decompensated heart failure, n	4 (18.2)	7 (31.8)	0.296
Acute coronary syndrome, n	2(9.1)	1 (4.5)	>0.99
Extrapulmonary sepsis, n	3 (13.6)	2 (9.1)	>0.99
Cardiac-thoracic surgery, n	1 (4.5)	1 (4.5)	>0.99
Others: emergency surgery, n	1 (4.5)	1 (4.5)	>0.99
Others, n	1 (4.5)	0	>0.99
Primary outcome			
72-hour therapy failure, n	4 (18.2)	2 (9.1)	0.664
Status for treatment failure within 72 hours			0.6
Hypoxemia respiratory failure, n	1	0	
Hypercapnia respiratory failure, n	3	1	
Excessive work of breathing, n	0	1	
Secondary outcome			
Reintubation within 72 hours, n	2 (9.1)	2 (9.1)	>0.99
Reintubation, n	5 (22.7)	7 (31.8)	0.498

ICU length of stay, days	14.5 (8-27)	12.5 (8-16)	0.217
Hospital length of stay, days	23.5 (19-42)	34 (22-49)	0.385
Hospital mortality, n	0	5 (22.7)	0.048*

Values are mean (standard deviation), median (interquartile range), or number (percentage)

APACHE II, Acute Physiology and Chronic Health Evaluation II; BMI, body mass index; COPD, chronic obstructive pulmonary disease; GCS, Glasgow Coma Scale; HFrEF, heart failure with reduced ejection fraction; ICU, intensive care unit; LVEF, left ventricular ejection fraction; MV, mechanical ventilation; Pemax, maximum expiratory pressure; Pimax, maximum inspiratory pressure; RSBI, rapid shallow breathing index; VHD, valvular heart disease.

* p value < 0.05
